# La ponction biopsie rénale: indications, complications et résultats

**DOI:** 10.11604/pamj.2018.31.44.15604

**Published:** 2018-09-20

**Authors:** Saad Alaoui Mhamedi, Hicham Meghraoui, Mohammed Benabdelhak, Yassamine Bentata, Intissar Haddiya

**Affiliations:** 1 Centre Hospitalier Universitaire Mohammed VI, Oujda, Maroc

**Keywords:** Ponction biopsie rénale, complications, néphropathie, technique, résultats histologiques, Renal needle biopsy, complications, nephropathy, technique, histological results

## Abstract

La ponction biopsie rénale (PBR) est une technique indispensable au diagnostic des maladies rénales. Ce geste demeure invasif avec diverses complications, essentiellement hémorragiques. L'objectif de notre travail était d'étudier notre pratique de la PBR, mettre le point sur la technique de la biopsie rénale transcutanée, évaluer ses complications, et déterminer les néphropathies diagnostiquées dans notre région. Il s'agit d'une étude rétrospective, menée entre Janvier 2015 et Juin 2017. Nous avons inclus 69 patients du service de Néphrologie du CHU Mohamed VI, Oujda, Maroc ayant bénéficié d'une biopsie de reins natifs. L'âge moyen des patients au moment de la PBR était de 38,16 ±13 ans (12-77 ans), le sexe ratio (H/F) était de 0,86. Le syndrome néphrotique était l'indication la plus fréquente tout âge confondu. Les néphropathies glomérulaires représentaient 81% des maladies rénales, réparties comme suit: la glomérulonéphrite extra-membraneuse retrouvée chez 19% des patients, la hyalinose segmentaire et focale chez 18%, la glomérulonéphrite lupique chez 11%, les glomérulonéphrites chroniques diagnostiquées dans 11% des cas, la glomérulonéphrite membrano-proliférative chez 7%, la glomérulonéphrite extra-capillaire chez 5% à part égale avec l'amylose rénale. Quatre pour cent (4%) des PBR étaient en faveur d'une néphropathie diabétique. Avec un taux estimé à 2% chacune, l'étude histopathologique avait retrouvé la glomérulonéphrite aigue post-infectieuse, la lésion glomérulaire minime, la néphropathie à IgA, les vascularites, les néphropathies tubulo-interstitielles ainsi que la microangiopathie thrombotique. Cinq pour cent (5%) des PBR étaient aglomérulaires. L'hématurie macroscopique était la principale complication observée dans notre série avec une fréquence de 2,8%. La PBR reste le gold standard du diagnostic de la maladie rénale, cependant le diagnostic syndromique permet au clinicien d'identifier la maladie rénale la plus probable et de guider les thérapeutiques urgentes.

## Abstract (en)

Renal needle biopsy (RNB) is the gold standard in the diagnosis of kidney diseases. It is an invasive technique causing several complications, in particular hemorrhagic events. This study aims to evaluate our RNB practice, to update the current understanding of the technique for percutaneous renal biopsy, to assess complications and to determine the prevalence of kidney diseases diagnosed in our region. We conducted a retrospective study between January 2015 and June 2017. Sixty-nine patients hospitalized in the Division of Nephrology at the University Hospital Mohamed VI Oujda having undergone native kidney biopsy were included in the study. The average age of patients, at the time of RNB, was 38.16 ±13 years [12-77 years], with a sex ratio (M/F) of 0.86. Nephrotic syndrome was the most frequent indication for RNB in all age groups. Glomerular kidney diseases accounted for 81% of kidney diseases, divided as follows: extra-membranous glomerulonephritis in 19% of patients, focal and segmental hyalinosis in 18%, lupus nephritisin in 11%, chronic glomerulonephrites in 11%, membranoproliferative glomerulonephritis in 7%, extracapillary glomerulonephritis in 5% shared equally with renal amyloidosis. Four percent of RNB showed diabetic nephropathy. Histopathological examination revealed acute post-infectious glomerulonephritis, minimal glomerular lesion, immunoglobulin A (IgA) nephropathy, vasculitis, tubulo-interstizial nephropathies as well as thrombotic microangiopathy, with an estimated rate of 2% respectively. Five percent of RNB showed non-glomerular disease. Macroscopic haematuria was the main complication observed in our case series, with a rate of 2.8%. RNB is the gold standard in the diagnosis of renal disease. However, syndromic diagnosis enables clinician to identify the most probable renal disease and to guide any emergency treatment.

## Introduction

La biopsie rénale marque une étape incontournable dans l'étude des maladies rénales. Elle fournit des arguments histologiques indispensables qui créent les bases de la nosologie des néphropathies, en particulier glomérulaires. Le marquage immunologique et les études de biologie moléculaire ont permis d'éclaircir l'individualisation clinique et histologique des maladies rénales et ont offert de nombreuses ouvertures à leur compréhension pathogénique. Iversen et Brun ont introduit la biopsie rénale percutanée en 1951 qui a subi plusieurs modifications par Kark et Muehrckc en 1954. Etant un geste invasif, la biopsie rénale peut avoir diverses complications essentiellement hémorragiques. Différentes études ont mis en évidence certains facteurs de risque de saignement. Cependant, les données de la littérature présentent d'importantes limites quant à la façon optimale de prédire et prévenir les complications hémorragiques après une biopsie rénale.

## Méthode

Nous avons mené une étude rétrospective, au sein du service de néphrologie du Centre Hospitalier Universitaire d'Oujda, Maroc, entre Janvier 2015 et Juin 2017. Les patients ont été sélectionnés à partir du registre de ponction biopsie rénale.

**Critères d'inclusion:** Nous avons inclus tous les patients ayant bénéficié d'une ponction biopsie du rein natif entre Janvier 2015 et juin 2017.

**Critères d'exclusion:** Ont été exclus de l'étude les patients hospitalisés dans les autres services, et les biopsies dont les renseignements cliniques sont manquantes sur les dossiers médicaux.

Nous avons relevé les éléments suivants: données épidémiologiques, cliniques, paracliniques des patients, les complications observées après la réalisation de la ponction biopsie rénale et les résultats histopathologiques. Les principaux syndromes ont été définis comme suit: **Syndrome néphrotique [SN]:** protéinurie supérieure à 3 g/j et hypo-albuminémie inférieure à 30 g/l; **Protéinurie non néphrotique isolée ou associée à une hématurie microscopique (PU/HU)**; **Syndrome néphritique aigu:** début brutal; une insuffisance rénale aiguë (IRA), intensité variable, hypertension artérielle (HTA) et une protéinurie parfois néphrotique; **Syndrome de glomérulonéphrite rapidement progressive (GNRP):** IRA d'aggravation rapide associée à une hématurie macroscopique inaugurale, l'absence habituelle d'HTA, et une protéinurie; **IRA isolée:** Elévation récente de la créatinine sérique, sans éléments en faveur de la chronicité. Nous avons utilisé le logiciel microsoft Exel 2010. Dans un premier temps, une analyse descriptive des caractéristiques épidémiologique, cliniques et biologiques des patients a été effectuée. Les résultats sont présentés sous forme de pourcentage et de moyenne ± Ecart type. Nous avons ensuite évalué le mode de présentation des différentes maladies rénales diagnostiquées par PBR.

## Résultats

Soixante-neuf PBR ont été réalisées entre 2015 et 2017. Dix huit PBR étaient réalisées en 2015, 37 en 2016 et 14 en 2017. L'âge moyen de nos patients au moment de la PBR était de 38,16±13 ans, avec des extrêmes allant de 12 à 77ans. Une légère prédominance féminine a été noté avec un sexe ratio [H /F] de 0.86. 40 de nos patients présentaient des antécédents répartis comme suit:1)17% avaient une HTA chronique sous traitement médical ; 2)11% des patients étaient suivis pour une néphropathie; 3)14% étaient suivis pour lupus; 4)La néphropathie était révélée chez nos malades par des signes rénaux différents à type de:1)syndrome œdémateux dans 60% des cas; 2) l'hypertension artérielle dans 36% des cas; 3) hématurie microscopique dans 56% des cas ; 4)hématurie macroscopique dans 2,8% des cas; 5) syndrome néphrotique dans 61% des cas. La protéinurie de 24 heures était d'ordre néphrotique dans 58% des cas, inférieure à 0,3g/24h dans 4,6% des cas, comprise entre 0,3 et 3g/24h dans 37% des cas.

**Bilan pré-biopsie**: Le Tableau 1 regroupe les résultats des bilans biologiques pré biopsie réalisés chez nos patients. Tous nos patients ont bénéficié d'échographie rénale qui n'a pas montré de contre- indication à la PBR. Les indications de la PBR étaient dominées par Le syndrome néphrotique (60%), tout âge confondu. La présence d'une maladie de système avec protéinurie ou atteinte rénale (14%) représentait la deuxième indication de la PBR avec l'IRA (14%), suivie par le syndrome de PU/HU (6%) GNRP chez (3%). Concernant la technique, la totalité des biopsies étaient percutanées, réalisées par un pistolet automatique type BARD avec aiguille de 16 Gauge à usage unique. Au cours ou suite au geste de biopsie rénale, différentes complications peuvent survenir, allant d'une simple hématurie jusqu'à la néphrectomie d'hémostase voire le décès. 2 de nos patients soit 2.8% (un homme et une femme) ont présenté une hématurie macroscopique. Ils ont bénéficié d'une échographie rénale. Pour la patiente elle a montré une lame d'épanchement sous capsulaire rénale gauche. En revanche, l'échographie était normale chez l'autre patient. Il n'y avait pas de différence significative d'âge, de sexe, ou dans bilan biologique ou bilan de coagulation avant la biopsie, entre patients avec et sans complications. Toutes les biopsies ont été étudiées en microscopie optique et en immunofluorescence. Les néphropathies glomérulaires représentaient 81% des maladies rénales diagnostiquées par PBR dans notre étude. La lésion glomérulaire minime (LGM): 2%, la hyalinose segmentaire et focale (HSF): 18%, la glomérulonéphrite extra-membraneuse (GEM): 19%, la glomérulonéphrite aigue post-infectieuse (GNA): 2%, la glomérulonéphrite membrano-proliférative (GNMP): 7%, la néphropathie à IgA (N IgA): 2%, la glomérulonéphrite extra-capillaire (GNEC): 5%, la glomérulonéphrite lupique (GL): 11%. Les glomérulonéphrites chroniques (GNC) ont été diagnostiquées dans 11% des cas. L'amylose rénale: 5%, la néphropathie diabétique: 4%, les vascularites: 2%. La PBR a révélé une néphrite tubulo-interstitielle aigue (NTIA) chez 2% des malades. La microangiopathie thrombotique était notée chez 2% des patients. 5% des PBR étaient aglomérulaires. La GEM était la principale cause du syndrome néphrotique 31% suivie par la HSF 18%, la GL 15% puis l'amylose 10%, GNMP 6%. Au dernier rang et à parts égales 3% venaient: la LGM, la GNEC, la GNA, la néphropathie diabétique. Concernant la GEM, elle s'est manifestée quasi-exclusivement par un syndrome néphrotique dans toutes les tranches d'âge. Suivie par le lupus qui est venu en deuxième rang avec une fréquence estimée à 9%.

**Tableau 1:** Bilan de nos patients avant la PBR

Bilan	Moyenne	Extrêmes
Hémoglobine (g/dl)	11,8	6,7-16,8
Plaquettes (/mm^3^)	316460	160000-810000
TP(%)	88,9	70-100
TCA	1,13	1-1.68

## Discussion

La pratique de la PBR en néphrologie clinique manque considérablement dans de nombreux pays en voie de développement et particulièrement en Afrique. Le service de Néphrologie du CHU de OUJDA est parmi les établissements hospitaliers qui pratiquent la PBR au niveau national. Nous avons répertorié 69 PBR sur une période de 2 ans et demi avec une moyenne de 27 PBR/an, cette fréquence est comparable à celle de H. Traore et *al*. [1]. Cette incidence annuelle était 103/an dans l'étude menée par Belarbia et *al.* [2] et 103/an dans l'étude menée par Mbarki et *al*. [3]. Cette incidence était plus importante dans Lei-shi et *al*. [4] 13519/an. L'âge moyen de nos patients au moment de la PBR était de 38,16±13 ans, ce qu'est comparable avec d'autres études menées en Afrique[5] et en Europe [6] Pour les manifestations rénales, l'HTA était retrouvée chez presque la moitié des patients (36%) et la réduction de la diurèse chez seulement 2%. Le dosage pondéral de l'excrétion urinaire des protéines de 24 heures a montré une protéinurie néphrotique chez 58% des patients, l'hématurie microscopique était retrouvée chez 58% d'entre eux, ainsi qu'une présence d'une insuffisance rénale au moment de la PBR chez 36% des malades. Ce qui est de même pour Mbarki et *al*. [5] et Raychlik et *al*. [7]. Le syndrome néphrotique est le mode de présentation le plus fréquent des maladies rénales chez nos patients de tout âge. Il représente ainsi la première indication de la PBR avec une fréquence de 61%. Ce résultat est comparable à celui retrouvé à Fès [5] avec une fréquence de 58,6%, en Espagne, cette fréquence était estimée à 31% [8] et 30% en Italie [9]. La présence d'une IRA est la deuxième indication de la PBR dans notre série 14%. C'est le cas également dans une étude menée en France avec une fréquence similaire (14%) [10]. La majorité des complications sont spontanément résolutives (hématurie, hématome périrénal de petite taille, fistule artérioveineuse. Des complications bénignes surviennent dans 13% des cas. Les complications majeures sont celles qui sont cliniquement significatives. Elles nécessitent une intervention thérapeutique (transfusion de culots globulaires (nécessaire dans 0,3 à 6% des cas), radiologie interventionnelle pour embolisation, néphrectomie…). Elles causent également une IRA, une septicémie voire le décès. La littérature rapporte que ces complications majeures surviennent dans 6 à 7% des biopsies rénales. Il n'existe pas de facteurs prédictifs de complications majeures. Les techniques d'embolisation ont réduit énormément le recours à la néphrectomie d'hémostase.

Les complications létales sont aussi devenues exceptionnelles [11]. Le Tableau 2 illustre les complications notables dans la littérature Internationale et les résultats dans notre série. Dans notre étude on n'a pas trouvé de relation entre les caractéristiques biologiques des patients et la survenue des complications. Par ailleurs Williams et *al* [11] ont constaté que les patients ayant une insuffisance rénale modérée ont un double risque de présenter une complication par rapport à ceux avec une fonction rénale normale après ajustement des troubles de la coagulation et la thrombopénie, et que les complications étaient deux fois plus fréquentes chez les patients avec une insuffisance rénale avancée. Pour notre série les complications étaient d'ordre de 2,8%. Les biopsies ont été réalisées par l'aiguille de 16 gauges. Existe-il un lien entre la taille de l'aiguille et les complications post-biopsie? Des études ont montré que le taux de transfusion était plus élevé chez les patients biopsiés avec une aiguille automatisée de calibre 14 par rapport aux aiguilles de calibre 16 et 18 [12], et que les aiguilles 16G entraînent moins d'hématomes post-biopsie et ont un rendement équivalent par rapport aux aiguilles de 14G [13]. Cependant, dans l'essai prospectif de Nicholson et *al*. [14] , aucune différence n'était observée entre les taux de complications pour les biopsies réalisées avec des aiguilles automatisées de 14, 16 ou 18 gauges. De même, d'autres études ont prouvé que la taille de l'aiguilles n'a pas d'influence sur le taux de complications ou bien les besoins transfusionnel des patients 9 et 10 [14-17]. Notre rapport a montré que les résultats anatomopathologiques avaient objectivé une néphropathie glomérulaire dans 81%, Elles représentent la première cause des maladies rénales prouvées par biopsie, ce qui est similaire à d'autres études [5, 18-20]. La GEM (19%) était la lésion glomérulaire la plus fréquente dans notre série. Une fréquence similaire a également été signalée en France (27%) [12], mais plus bas en Espagne à 24% [10] . Pour d'autres études, la HSF vient en premier, c'est le cas de l'étude de Polito et *al*. [11]. la HSF était en deuxième position avec une fréquence de (18%), ce résultat est concordant avec Naumovic et *al*. [8] . Chez l'ensemble de nos patients, les premières causes du syndrome néphrotique sont la GEM, et la HSF et GL. Ceci a été retrouvé également dans d'autres études [5]. Chez le sujet âgé, c'est la GNC qui était la plus fréquente. Ces résultats sont différents de ceux rapportés par Ben Kaab et *al*. [21], cette discordance pourrait être expliquée par le retard de réalisation de la PBR, lié le plus souvent à la consultation tardive chez notre population, ainsi qu'à l'absence d'un bilan antérieur pouvant orienter vers le caractère aigu ou chronique de la maladie rénale [5]. Le diagnostic histologique est d'une grande importance en néphrologie clinique. Il permet, en effet, de guider le traitement approprié, en particulier en cas de vascularite rénale et de GNEC qui représentent les grandes urgences néphrologiques diagnostiques et thérapeutiques.

**Tableau 2:** Les complications notables dans la littérature internationale et les résultats dans notre série

Complications PBR	Nombre des cas	Prévalence (%)	Nombre des cas	Prévalence (%)
Hématurie macroscopique	23	3,1	2	2,8
Hématome périrénale	16	2,1	0	0
Fistule Artérioveineuse	3	0,4	0	0
Décès	2	0,2	0	0

## Conclusion

La biopsie rénale est une technique indispensable au diagnostic des maladies rénales primitives et secondaires. Deux modifications techniques majeures ont augmenté significativement sa sécurité et son efficacité: l'échoguidage en temps réel et l'utilisation du pistolet automatique, et qui ont été utilisées dans notre étude. On n'a pas noté de complications majeures. Sauf les complications mineurs, représentaient par l'hématurie microscopique ont été observées. Le syndrome néphrotique correspond au premier mode de présentation clinique des maladies rénales, la GEM, la HSF et la GL sont les premières causes. Le diagnostic syndromique permet au clinicien d'identifier la maladie rénale la plus probable et de guider les thérapeutiques urgentes en attendant les résultats de la PBR. Cependant, cette approche ne doit pas être considérée comme une alternative à la PBR qui reste le gold standard du diagnostic de la maladie rénale.

### Etat des connaissances actuelles sur le sujet

- La PBR marque une étape incontournable dans l'étude des maladies rénales;
- Elle fournit des arguments histologiques indispensables qui créent les bases de la nosologie des néphropathies;
- C'est un geste invasif et par conséquent il peut y avoir différentes complications.

### Contribution de notre étude à la connaissance

- Les résultats histologiques de la PBR dans notre pays;
- Les complications de la PBR dans notre contexte;
- Intérêt de la prévention de ces complications.

## Conflits d’intérêts

les auteurs ne déclarent aucun conflit d'intérêts.
